# Editorial: Exploring molecular mechanisms in cancer through tumor molecular pathology

**DOI:** 10.3389/fonc.2026.1881199

**Published:** 2026-05-26

**Authors:** Wenbo Ma, Haifeng Zhang, Nianli Liu, Caiyue Li

**Affiliations:** 1 Department of Hepatobiliary Surgery, The Eighth Affiliated Hospital, Southern Medical University (The First People’s Hospital of Shunde Foshan), Guangdong, China; 2 Key Laboratory of Tumor Microenvironment Regulation of Guangdong Higher Education Institutes, The Eighth Affiliated Hospital, Southern Medical University (The First People’s Hospital of Shunde Foshan), Guangdong, China; 3 Foshan Clinical Technology Innovation Base of Traditional Chinese Medicine for Integrated Traditional Chinese and Western Medicine in Cancer Prevention and Treatment, The Eighth Affiliated Hospital, Southern Medical University (The First People’s Hospital of Shunde Foshan), Guangdong, China; 4 Foshan Engineering Technology Research Center of Molecular Medicine, The Eighth Affiliated Hospital, Southern Medical University (The First People’s Hospital of Shunde Foshan), Guangdong, China; 5 Shunde Regional Medical Research Center, The Eighth Affiliated Hospital, Southern Medical University (The First People’s Hospital of Shunde Foshan), Guangdong, China; 6 Department of Pathology, School of Basic Medical Sciences, Xi’an Jiaotong University Health Science Center, Xi’an Jiaotong University, Xi’an, China; 7 Cancer Institute, Cellular Therapeutics School of Medicine, Xuzhou Medical University, Xuzhou, Jiangsu, China; 8 Medical Research Center, The Eighth Affiliated Hospital, Southern Medical University (The First People’s Hospital of Shunde Foshan), Guangdong, China

**Keywords:** biomarkers, cancer, molecular diagnostics, precision oncology, targeted therapy, tumor microenvironment, tumor molecular pathology

Tumor molecular pathology has become an indispensable discipline for understanding cancer initiation, progression, therapeutic response, and clinical heterogeneity (1). By integrating histopathological assessment with genomic, transcriptomic, proteomic, immunological, and functional analyses, this field bridges morphological diagnosis with molecularly informed clinical decision-making (1, 2). As cancer treatment increasingly shifts toward precision oncology, molecular pathology provides the conceptual and technical foundation for identifying actionable alterations, refining prognostic stratification, guiding targeted therapy and immunotherapy, and uncovering novel mechanisms of tumor evolution and treatment resistance (1, 3, 4).

This Research Topic, “Exploring Molecular Mechanisms in Cancer through Tumor Molecular Pathology,” was established to provide a comprehensive platform for studies that investigate the molecular basis of tumor behavior and translate these insights into diagnostic, prognostic, and therapeutic advances. The Research Topic brings together 19 articles, including 3 reviews, 12 original research articles, 2 case reports, 1 brief research report, and 1 technology and code article. Together, these contributions highlight the breadth of tumor molecular pathology, spanning cancer-associated viral oncogenesis, molecular diagnostics, rare genomic alterations, immune profiling, cell signaling, apoptosis regulation, therapeutic resistance, and biomarker-driven precision treatment.

Several articles in this Research Topic focus on molecular mechanisms that drive tumor development and progression. Xie et al. reviewed Epstein–Barr virus-driven molecular pathogenesis in primary pulmonary lymphoepithelial carcinoma, emphasizing the links among viral infection, tumor immune microenvironment, clinicopathological features, and potential EBV-targeted or immunotherapeutic strategies. Samaha et al. summarized the molecular pathogenesis of adult T-cell leukemia/lymphoma, highlighting the contributions of HTLV-1 infection, viral oncogenes, immune escape, and recurrent alterations in TCR/NF-κB, JAK/STAT, and apoptotic pathways. Sun et al. reviewed the Hippo signaling pathway in cervical cancer, particularly the crosstalk between HPV oncoproteins and YAP/TAZ-mediated transcriptional programs, and discussed its therapeutic potential. These reviews collectively provide conceptual frameworks for understanding how viral factors, signaling networks, and tumor-specific molecular dependencies shape malignant phenotypes.

A group of original studies further expands the mechanistic landscape of cancer molecular pathology. Chen et al. demonstrated that ZC3H18 regulates alternative splicing events and cancer-associated pathways in cervical cancer, suggesting that RNA-binding proteins and splicing regulation may represent important therapeutic vulnerabilities. Chen et al. identified NOP56 as an oncogenic nucleolar protein in hepatocellular carcinoma that interacts with fibrillarin and activates the PI3K/AKT/CREB pathway to inhibit apoptosis. Yan et al. showed that endoplasmic reticulum stress-induced CLGN promotes apoptosis resistance in hepatocellular carcinoma through NF-κB signaling, while paeonol synergistically enhances antitumor efficacy by dual inhibition of CLGN and NF-κB. Lu et al. reported that CMSP suppresses oral squamous cell carcinoma progression by targeting the JAK2/STAT3/c-Myc axis. Chen et al. provided experimental evidence that HOXB7 drives bladder cancer progression through H-Ras/Raf-1/MEK/ERK signaling. These studies underscore the importance of pathway-level molecular pathology in identifying functional drivers and candidate therapeutic targets.

Another major theme is the clinical application of molecular diagnostics and biomarker discovery. Mohamed et al. showed that combined PRSS22 and CEA mRNA analysis in lymph nodes can identify the majority of colon cancer patients who relapse within 12 years, supporting PRSS22 as a promising prognostic biomarker and potential therapeutic target. Zhang et al. found that TP53 and KRAS co-mutations are associated with worse outcomes in mucinous ovarian carcinoma, suggesting that tumor molecular profiling may improve prognostic assessment even in early-stage disease. Conca et al. demonstrated the clinical utility of genomic instability metrics and CCNE1 amplification in stratifying high-grade serous ovarian cancer, particularly among homologous recombination-proficient patients. Man et al. identified APOL1 as a prognostic biomarker in thyroid cancer and linked its expression to immune infiltration, illustrating the value of integrating molecular markers with tumor immune contexture.

Several articles address rare or clinically challenging molecular-pathological entities. Srivastav et al. described metastatic colorectal cancer cases harboring concomitant BRAF and RAS family mutations, a rare molecular subset with important implications for treatment interpretation and future research. Gao et al. reported two cases of bronchiolar adenoma/ciliated muconodular papillary tumor with prominent basal cell hyperplasia and squamous metaplasia, highlighting diagnostic pitfalls and the importance of immunohistochemistry and molecular testing. Guo et al. presented a rare case of metastatic INI-1-deficient undifferentiated lung cancer with an EGFR exon 19 deletion identified in pleural effusion, expanding current knowledge of SWI/SNF-deficient intrathoracic tumors.

Importantly, this Research Topic also highlights how molecular pathology can inform treatment selection and therapeutic optimization. Evrard et al. used patient-derived xenograft models to prospectively model mutation-based therapeutic strategies from the NCI-MPACT trial, showing that temozolomide sensitivity correlated with MGMT deficiency across cancer types. Kong et al. reported that early brain radiotherapy combined with third-generation EGFR-TKIs improved intracranial progression-free survival and overall survival in EGFR-mutant non-small cell lung cancer with synchronous brain metastases. Boeckaerts et al. investigated efferocytosis modulation in intestinal epithelial cells during colorectal cancer, providing important negative findings that refine future experimental directions. Rangarajan et al. developed a structure-based tool to interpret kinase variants of unknown significance in clinical next-generation sequencing, illustrating how structural biology can complement molecular diagnostics and support precision oncology.

Collectively, the studies in this Research Topic highlight the expanding role of tumor molecular pathology in bridging molecular mechanisms with translational and clinical oncology. By integrating molecular profiling, functional validation, biomarker discovery, computational analysis, and therapeutic exploration, these works provide deeper insights into tumor heterogeneity, immune regulation, signaling pathways, and therapy resistance across multiple cancer types. Importantly, this Research Topic reflects emerging trends in cancer research, including multi-omics integration, AI-assisted molecular diagnostics, structure-guided precision medicine, and personalized therapeutic strategies. Beyond advancing mechanistic understanding, these studies also emphasize the growing importance of translating molecular discoveries into clinically actionable applications. We anticipate that the findings presented here will promote interdisciplinary collaboration and contribute to the development of more accurate diagnostic tools, more effective targeted therapies, and more individualized treatment paradigms in precision oncology.
